# Assessment of liver fibrosis by transient elastography in young children with chronic hepatitis B virus infection

**DOI:** 10.1007/s12072-021-10194-7

**Published:** 2021-07-09

**Authors:** Zhiqiang Xu, Jinfang Zhao, Jiaye Liu, Yi Dong, Fuchuan Wang, Jianguo Yan, Lili Cao, Pu Wang, Aiqin Li, Jing Li, Shishu Zhu, Yanwei Zhong, Min Zhang, Fu-Sheng Wang

**Affiliations:** 1 Medical School of Chinese PLA, Beijing, China; 2 Department of Liver Disease of Chinese, Fifth Medical Center of Chinese, PLA General Hospital, PLA General Hospital, Beijing, China; 3 Treatment and Research Center for Infectious Diseases, Fifth Medical Center of Chinese, PLA General Hospital, 100 Western 4th Ring Road, Beijing, 100039 China

**Keywords:** Young children, Chronic hepatitis B, LSM, Fibrosis, Liver biopsy

## Abstract

**Background:**

This study aimed to compare the diagnostic accuracy of transient elastography (TE) and biopsy for the detection of liver fibrosis in children with chronic hepatitis B (CHB).

**Methods:**

This single-center prospective study included 157 CHB children aged 0–6 years. All patients underwent liver stiffness measurement (LSM) by TE and liver biopsy, separated by an interval of less than 1 week.

**Results:**

The LSM, aspartate aminotransferase-platelet ratio index (APRI), and fibrosis-4 index (FIB-4) were positively correlated with activity grade and fibrosis stage in CHB children. The areas under the receiver operating characteristic curves (AUCs) of LSM for identifying significant (F ≥ 2) and advanced (F ≥ 3) fibrosis were 0.732 and 0.941, respectively. The cut-off values, specificity, and sensitivity for significant fibrosis were 5.6 kPa, 75.7%, and 67.4%, respectively; the corresponding values for advanced fibrosis were 6.9 kPa, 91.5%, and 81.3%, respectively. Compared to LSM, the overall diagnostic performances of APRI and FIB-4 for significant and advanced fibrosis were suboptimal, with low AUCs and sensitivity. Since LSM, platelet, and Log_10_ (hepatitis B surface antigen) were independent factors associated with the fibrosis stage (F < 2 and F ≥ 2), they were used to formulate the “LPS” index for the prediction of F ≥ 2. The AUC of LPS (for F ≥ 2) was higher than that of LSM (0.792 vs. 0.732, *p* < 0.05), and had an improved sensitivity (76.6% vs. 67.4%).

**Conclusions:**

TE is a promising technology for the diagnosis of advanced fibrosis in CHB children aged 0–6 years.

**Supplementary Information:**

The online version contains supplementary material available at 10.1007/s12072-021-10194-7.

## Introduction

Hepatitis B virus (HBV) infection is one of the most common causes of chronic liver disease worldwide, especially in China where more than 80 million adults and 37,000 children are affected [1, 2]. Although the natural history of chronic HBV infections in children remains poorly understood, a limited number of studies have shown that 1–5% of hepatitis B e antigen (HBeAg)-positive children develop cirrhosis before adulthood [3–6]. In addition, 25% of adult patients who acquire HBV infection in childhood will develop liver cancer or cirrhosis, both of which are associated with high morbidity and mortality [7]. Thus, there is a critical need to decrease the risk of disease progression to cirrhosis, and develop a functional cure for chronic hepatitis B (CHB). One of the most important indicators for antiviral treatment is histological evidence of necro-inflammation and fibrosis, according to the guidelines of the European Society of Paediatric Gastroenterology, Hepatology, and Nutrition (ESPGHAN), as well as the American Association for the Study of Liver Diseases [8]. Therefore, the early diagnosis of the extent of liver inflammation and fibrosis is important for the treatment of CHB during childhood [8, 9].

Currently, liver biopsy remains the gold standard for determining the degree of liver inflammation and fibrosis and is integral for guiding antiviral treatment in children with CHB [8, 9]. Nevertheless, follow-up biopsies are required to evaluate the efficacy of antiviral treatment [10]; these procedures are associated with pain, additional expenses, and risks of post-procedure hospitalization [11]. Moreover, a liver biopsy requires highly skilled physicians and medical devices. Thus, there is a need for the development of non-invasive tests to diagnose liver cirrhosis in children with CHB, in order to avoid the risks and costs associated with liver biopsies.

The aspartate aminotransferase (AST)-platelet (PLT) ratio index (APRI) and fibrosis-4 index (FIB-4) scores, obtained by evaluating laboratory parameters, have been used to identify fibrosis stages in adult patients with CHB. However, a previous study has shown that the APRI and FIB-4 are inadequate due to high rates of misclassification [12]; the diagnostic performance of the APRI and FIB-4 in children with CHB remains unknown. Transient elastography (TE) is a novel noninvasive assessment tool that has been widely adopted to diagnose liver fibrosis stage and monitor the development of chronic liver diseases (e.g., CHB and chronic hepatitis C [CHC]) in adult patients, due to its accuracy and reproducibility [13]. Several studies have shown that the liver stiffness measurement (LSM) determined via TE is useful for the assessment of liver fibrosis in children with chronic liver disease [14, 15]. Although the LSM has been used to evaluate hepatitis B- and C-related fibrosis in children across three studies, their results have been limited by small sample sizes, restricted patient populations (primarily adolescents and young adults), as well as the inclusion of liver diseases due to multiple causes [11, 15, 16]. To date, no studies have assessed the effectiveness of TE in the detection of liver fibrosis in children with CHB. Therefore, the purpose of this study was to compare the diagnostic accuracy of TE and biopsy for the detection of liver fibrosis in children with CHB.

## Patients and methods

### Patient recruitment

This prospective study enrolled 157 CHB patients (aged 0–6 years) from June 2015 to March 2020 at Fifth Medical Center of Chinese PLA General Hospital. Patients were included if they were aged ≤ 6 years; met the criteria for CHB, according to the guidelines for prevention and treatment of CHB in China [17]; and underwent LSM and liver biopsy, separated by an interval of less than 1 week. and written informed consent was obtained from the parent or legal guardian of the child subjects. The exclusion criteria comprised the following: (1) white blood cells < 2.75 × 10^9^/L, PLT < 80 × 10^9^/L, total bilirubin > 51 μmol/L, alanine aminotransferase (ALT) ≥ 400 IU/L, serum creatinine > 133 μmol/L, or international normalized ratio > 1.5; (2) patients positive for hepatitis A/C/delta virus, human immunodeficiency virus, or a chronic liver disease other than CHB (e.g., autoimmune hepatitis, Wilson’s disease, hepatolenticular degeneration, and hepatocellular carcinoma); (3) evidence of decompensation (i.e., clinical ascites); and (4) any other serious physical and mental illnesses. Written informed consent was obtained from the parent or legal guardian of all child patients.

### Clinical and laboratory parameters

Demographic data, including age, gender, body weight, and height (Body Mass Index [BMI] = body weight in kg/[height in meters]^2^), were collected. Routine blood tests, liver function tests, abdominal ultrasound examination, and plasma HBV deoxyribonucleic acid (DNA) and serological HBV marker (including HBeAg and hepatitis B surface antigen [HBsAg]) quantification were performed. The APRI and FIB-4 were calculated by using the following formula, as previously reported [12]: APRI = [AST level (IU/L)/AST upper level of normal (IU/L)]/PLT count (10^9^/L) × 100 and FIB-4 = [age (years) × AST level (IU/L)]/[PLT count (10^9^/L) × ALT level (IU/L)].

### Liver histology and LSMs

After the laboratory examinations were performed, ultrasonic-guided liver biopsies were carried out in all patients using a 1-s needle biopsy. Liver specimens were prepared for histological evaluation by a senior pathologist, who was blinded to the LSM results, according to the meta-analysis of histological data for viral hepatitis (METAVIR) scoring system [18]. The LSM was expressed in kPa, and measured by a certified and experienced physician (blinded to the liver biopsy results) using the Fibroscan^®^ and S probe (Echosens, France). The LSM results were only considered to be reliable when an interquartile range (IQR)/LSM of ≤ 0.3 was obtained, across a maximum of 10 validated measurements.

### Statistical analysis

Quantitative variables are expressed as the mean ± standard deviation or median and IQR; categorical variables are expressed as the number and percentage of patients. Quantitative variables were compared using the Student *t* test/one-way analysis of variance for normally distributed variables, or Tamhane’s T2 test for non-normally distributed variables. Categorical variables were compared using the Chi-squared test. Correlations were assessed using Spearman's rank correlation coefficient, and factors associated with the degree of liver fibrosis were identified with a logistic regression analysis. The diagnostic value of the LSM was evaluated based on the following: sensitivity; specificity; positive and negative predictive values; positive and negative likelihood ratios; and the area under the receiver operating characteristic (ROC) curve (AUC), as determined via the Hanley-McNeil test. The LSM cut-off values for predicting the different stages of liver fibrosis were determined at the highest sensitivity and specificity. All statistical analyses were performed using SPSS 25.0 statistical software (Armonk, NY, USA). The level of statistical significance was set at *p* < 0.05.

## Results

### Patient demographic and laboratory variables

Among the 157 included patients, 92 (58.6%) were male; the median age was 3.0 years (IQR, 1.9, 4.1), and the median BMI was 16.01 (15.00, 17.28) (Table 1). The laboratory variables, including the white blood cell count (8.2 ± 2.1, 10^9^/L), PLT count [287 (236, 344), 10^9^/L], ALT level [73 (42, 145), IU/L], AST level [79 (55, 136), IU/L], Log_10_ HBV DNA quantification [7.87 (7.00, 8.01), IU/mL], and serological HBV markers are summarized in Table 1.

**Table 1:** Patient variables

Variable	Patients (*n* = 157)
Male gender, *n* (%)	92 (58.6)
Age (median, IQR, years)	3.0 (1.9, 4.1)
BMI (median, IQR, kg/m^2^)	16.01 (15.00, 17.28)
ALT (median, IQR, IU/L)	73 (42, 145)
AST (median, IQR, IU/L)	79 (55, 136)
Total bilirubin (median, IQR, μmol/L)	6.2 (4.9, 8.3)
ALP (median, IQR, IU/L)	283 (233, 338)
γ-GT(median, IQR, IU/L)	17 (13, 29)
Albumin (median, IQR, g/L)	41 (39, 43)
cholinesterase (median, IQR, IU/L)	7933 ± 1644
WBC count (means ± SD, 10^9^/L)	8.2 ± 2.1
PLT count (median, IQR, 10^9^/L)	287 (236, 344)
HBeAg postitive, *n* (%)	143 (91.1)
HBeAg (median, IQR, COI)	1457 (435, 1814)
HBsAg quantification (median, IQR, IU/mL)	18,101 (5133, 42,793)
Log_10_ HBsAg (median, IQR, IU/mL)	4.26 (3.71, 4.63)
Log_10_ HBV DNA (median, IQR, IU/mL)	7.87 (7.00, 8.01)
APRI (median, IQR)	0.6778 (0.4573, 1.1296)
FIB-4 (median, IQR)	0.0951 (0.0639, 0.143)
LSM (median, IQR, kPa)	5.2 (4.4–6.1)
Activity grade, *n* (%)	
A0	2 (1.3)
A1	57 (3.3)
A2	96 (61.1)
A3	2 (1.3)
Fibrosis stage, *n* (%)	
F0	16 (10.2)
F1	95 (60.5)
F2	30 (19.1)
F3	13 (8.3)
F4	3 (1.9)

*IQR* interquartile range, *BMI* body mass index, *ALT* alanine aminotransferase, *AST* aspartate aminotransferase, *ALP* alkaline phosphatase, *γ-GT* gamma-glutamyl transpeptidase, *WBC* white blood cell, *PLT* platelet, *HBeAg* hepatitis B e-antigen, *HBsAg* hepatitis B surface antigen, *LSM* liver stiffness measurement, *APRI* aspartate aminotransferase-to-Platelet ratio index, *FIB-4* the Fibrosis-4 score, *COI* cutoff index

### Noninvasive assessment indices for liver fibrosis and histological features

The LSM was 5.2 (4.4–6.1) kPa, ranging from 1.1 to 12.6 kPa (Table 1). The APRI and FIB-4 scores were 0.6778 (0.4573, 1.1296) and 0.0951 (0.0639, 0.1434), respectively. In addition, 59 patients presented with either mild or no necroinflammatory activity (A < 2); 96 and 2 patients were assigned scores of A2 and A3, respectively. Mild liver fibrosis, or a lack thereof (F0–F1), was observed in 111 patients; 30 patients had a score of F2, and 16 patients exhibited an advanced fibrosis (F ≥ 3) (Table 1).

### Correlation between LSM, APRI and FIB-4 with activity grade and liver fibrosis stage

**Fig. 1:**
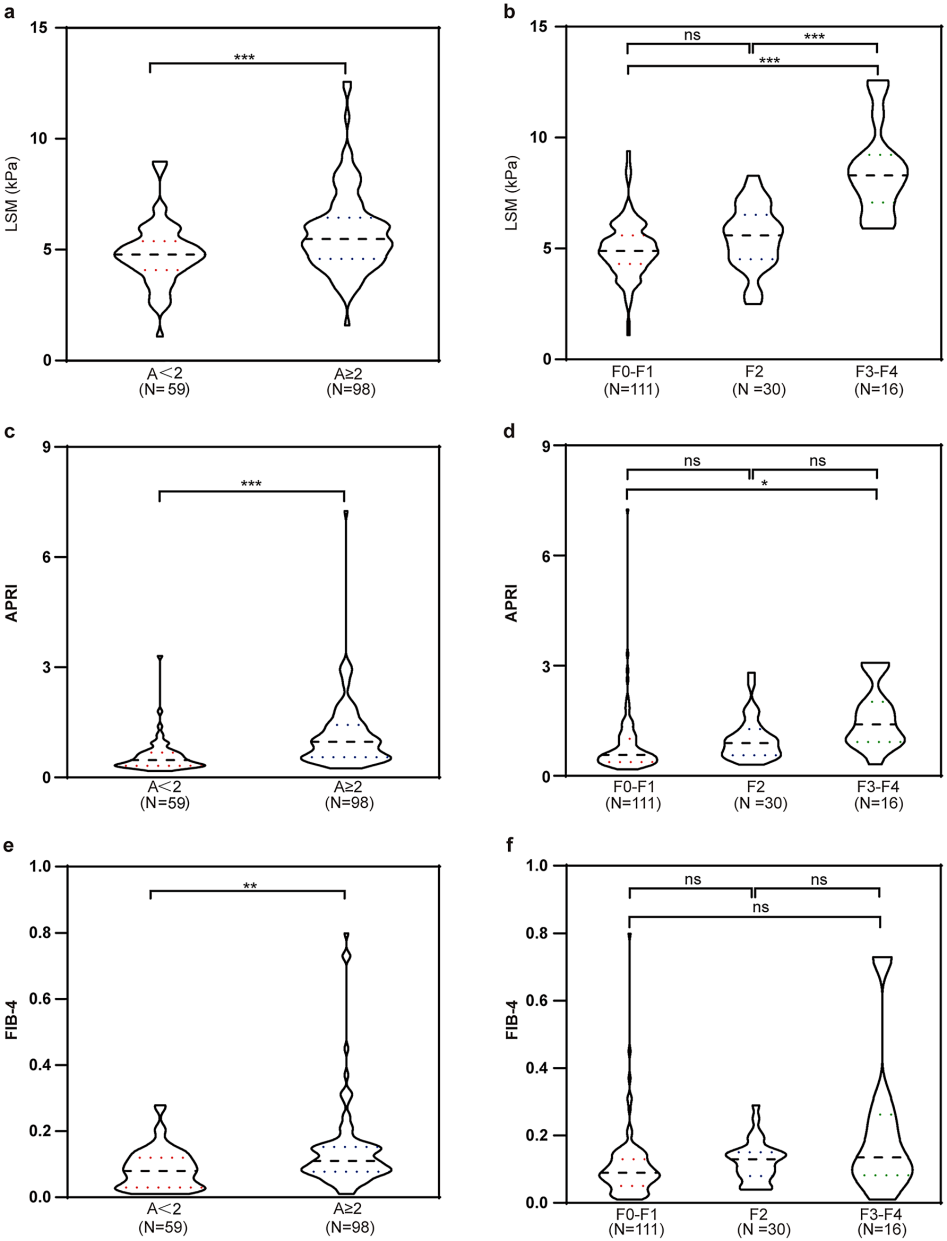
Correlation between LSM, APRI or FIB-4 and histological features in children with CHB. **a** LSM vs. activity grade (A < 2 and A ≥ 2). **b** LSM vs. METAVIR liver fibrosis stage (F0–F1, F2, and F3–F4). **c** APRI vs. activity grade (A < 2 and A ≥ 2). **d** APRI vs. METAVIR liver fibrosis stage (F0–F1, F2, and F3–F4). **e** FIB-4 vs. activity grade (A < 2 and A ≥ 2). **f** FIB-4 vs. METAVIR liver fibrosis stage (F0–F1, F2, and F3–F4). *p < 0.05; **p < 0.01; ***p < 0.001; *ns* no significant difference, *LSM* liver stiffness measurement, *kPa* kilopascal, *APRI* aspartate aminotransferase-to-platelet ratio index, *FIB-4* fibrosis-4 index, *METAVIR* meta-analysis of histological data for the viral hepatitis

The activity grades were divided into two groups (A < 2 and A ≥ 2), and liver fibrosis stages were classified into three groups (F0–F1, F2, and F3–F4), in accordance with previous studies [14]. The distribution of the LSM, APRI, and FIB-4 according to activity grade and stages of liver fibrosis are displayed in Fig. 1. A comparative analysis showed that the A ≥ 2 group [5.5 (4.6–6.5) kPa] had a higher median LSM value than that of the A < 2 group [4.8 (4.1–5.4) kPa] (*p* < 0.001) (Fig. 1a). Patients classified with the F3–F4 stage had a significantly higher LSM compared to those with the F0–F1 (8.3 vs. 4.9 kPa; *p* < 0.001) and F2 (8.3 vs. 5.6 kPa; *p* < 0.001) stage. There was no significant difference in LSM values between patients in the F0–F1 stage and F2 stage (Fig. 1b). In terms of the APRI, the A ≥ 2 group had higher values compared to the A < 2 group (0.9726 vs. 0.4664, *p* < 0.001). Patients classified with the F3–F4 stage had significantly higher values than those classified with the F0–F1 stage (1.4040 vs. 0.5662, *p* < 0.05) (Fig. 1c and d). In addition, FIB-4 levels were higher in the A ≥ 2 group compared to the A < 2 group (0.1104 vs. 0.0814, *p* < 0.01); however, there were no significant differences in fibrosis stages among the three groups (F0–F1, 0.0896; F2, 0.1321; and F3–F4, 0.1337; all *p* > 0.05) (Fig. 1e and f).

We subsequently estimated the correlations between clinical and histological parameters (LSM, APRI, and FIB-4) with both activity grades (A < 2 and A ≥ 2) and liver fibrosis stages (F0–F1, F2, and F ≥ 3). The LSM (*r* = 0.275, *p* < 0.001), APRI (*r* = 0.478, *p* < 0.001), and FIB-4 (*r* = 0.249, *p* < 0.01) were all positively correlated with the degree of activity. All three laboratory and histological parameters were also positively correlated with the degree of fibrosis (LSM, *r* = 0.414, *p* < 0.001; APRI, *r* = 0.357, *p* < 0.001; FIB-4, *r* = 0.277, *p* < 0.001). These results suggest that the LSM, APRI, and FIB-4 are positively associated with the severity of liver inflammation and fibrosis in children with CHB.

### Diagnostic value of the LSM, APRI, and FIB-4 for liver fibrosis stages

**Fig. 2:**
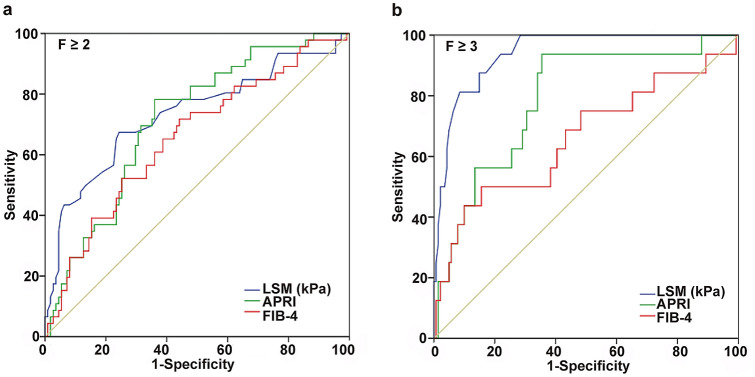
AUCs of LSM, APRI, and FIB-4 for the diagnosis of liver biopsy fibrosis stage. The AUCs of LSM, APRI, and FIB-4 for the **a** F ≥ 2 fibrosis stage and **b** F ≥ 3 fibrosis stage. *AUC* area under the receiver operating characteristic (ROC) curve, *LSM* liver stiffness measurement, *kPa* kilopascal, *APRI* aspartate aminotransferase-to-platelet ratio index, *FIB-4* fibrosis-4 score, *METAVIR* meta-analysis of histological data for viral hepatitis

To further evaluate the performance of the LSM, APRI, and FIB-4 for the liver fibrosis stages, ROC curve analysis was performed for all patients. The AUCs of the LSM for the identification of fibrosis stages F ≥ 2 and F ≥ 3 among children with CHB were 0.732 (95% confidence interval [CI], 0.639–0.826) and 0.941 (95% CI 0.897–0.985), respectively (Table 2). The optimal cut-off values, specificity, and sensitivity for F ≥ 2 were 5.6 kPa, 75.7% (95% CI 66.6–83.3), and 67.4% (95% CI 52.0–80.5), respectively; the corresponding values for F ≥ 3 were 6.9 kPa, 91.5% (95% CI 85.6–95.5), and 81.3% (95% CI 54.4–96.0), respectively (Table 2; Fig. 2a and b). While the specificities of the APRI for the prediction of F ≥ 2 and F ≥ 3 were moderately higher than those of the LSM, both the AUCs and sensitivities of the APRI and FIB-4 for F ≥ 2 and F ≥ 3 were lower, especially for F ≥ 3 (Table 2; Fig. 2a and b). These results suggest that the LSM is more reliable than the APRI and FIB-4 for the assessment of advanced liver fibrosis; nevertheless, all three of these parameters were suboptimal for the identification of significant liver fibrosis.

**Table 2:** The diagnostic performance of LSM, APRI, and FIB-4 for the identification of fibrosis stages

Fibrosis stage	Cutoff (kPa)	YI	Se (%, 95% CI)	Sp (%, 95% CI)	PLR (95% CI)	NLR (95% CI)	PPV (%, 95% CI)	NPV (%, 95% CI)	AUC (95% CI)
LSM									
F ≥ 2	5.6	0.43	67.4 (52.0–80.5)	75.7 (66.6–83.3)	2.3 (1.5–3.6)	0.36 (0.20–0.50)	84.8 (78.5–89.6)	53.4 (43.9–62.8)	0.732 (0.639–0.826)
F ≥ 3	6.9	0.73	81.3 (54.4–96.0)	91.5 (85.6–95.5)	4.9 (1.8–13.5)	0.10 (0.06–0.20)	97.7 (93.9–99.2)	52 (37.5–66.2)	0.941 (0.897–0.985)
APRI									
F ≥ 2	0.7159	0.4222	64.0 (54.3–72.9)	78.3 (63.6–89.1)	2.9 (1.7–5.2)	0.46 (0.30–0.60)	87.7 (80.1–92.6)	47.4 (40.2- 54.6)	0.713 (0.636–0.783)
F ≥ 3	0.8156	0.5829	64.5 (56.0–72.4)	93.7 (69.8–99.8)	10.3 (1.5–69.2)	0.38 (0.30–0.50)	98.9 (93.1–99.8)	23.1 (18.8–27.9)	0.790 (0.718–0.851)
FIB-4									
F ≥ 2	0.0928	0.2759	55.8 (46.1–65.3)	71.7 (56.5–84.0)	2.0 (1.2–3.2)	0.62 (0.50–0.80)	82.7 (74.5–88.6)	40.2 (33.8–47.0)	0.655 (0.575–0.729)
F ≥ 3	0.1543	0.3440	84.4 (77.3–90.0)	50.0 (24.7–75.3)	1.7 (1.0–2.8)	0.31(0.20–0.60)	93.7 (90.1–96.1)	26.7 (16.3–40.4)	0.660 (0.580–0.734)

*Se* sensitivity, *Sp* specificity, *PLR* positive likelihood ratio, *NLR* negative likelihood ratio, *PPV* positive predictive values, *NPV* negative predictive values, *AUC* area under receiver operating characteristics (ROC) curves, *LSM* liver stiffness measurement, *APRI* aspartate aminotransferase-to-Platelet ratio index, *FIB-4* Fibrosis-4 score

### Parameters independently associated with the F ≥ 2 fibrosis stage

A univariate analysis was performed to assess the potential associations between the fibrosis stage and both clinical and laboratory parameters (Table S1). The results indicated that ALT, AST, gamma-glutamyl transpeptidase, cholinesterase, PLT, HBeAg, HBsAg, Log_10_HBsAg, Log_10_HBV DNA, A ≥ 2, and LSM were significantly associated with the fibrosis stage (F ≥ 2) (all *p* < 0.05) (Table S1). LSM, PLT, and Log_10_HBsAg remained significantly associated with the fibrosis stage in the multivariate analysis (all *p* < 0.05) (Table 3).

**Table 3:** Independent factors associated with the fibrosis stages of the liver biopsy

	Estimate ± SE	Odds ratio (95% CI)	*p*
LSM (kPa)	0.511 ± 0.131	1.667 (1.289 ~ 2.155)	< 0.001
PLT (10^9^/L)	− 0.006 ± 0.003	0.994 (0.988 ~ 0.999)	0.032
Log_10_ HBsAg (IU/mL)	− 0.682 ± 0.258	0.505 (0.305 ~ 0.837)	0.008

*SE* standard error, *CI* confidence interval, *PLT* platelet, *LSM* liver stiffness measurement, *HBsAg* hepatitis B surface antigen

### Combination of LSM, PLT, and Log_10_HBsAg for the determination of the F ≥ 2 liver fibrosis stage

Since LSM had a relatively poor diagnostic accuracy for F ≥ 2 (Table 2; Fig. 2), LSM, PLT, and Log_10_HBsAg were combined as independent factors to create an algorithm for the prediction of the F ≥ 2 liver fibrosis stage. This algorithm was referred to as the LPS index: LSM, PLT, and Log_10_HBsAg = 0.511 × LSM − 0.006 × PLT − 0.682 × Log_10_HBsAg + 0.769. The results indicated that the AUC increased to 0.792 (95% CI 0.720–0.852), which was higher than that of LSM (0.792 vs. 0.732, *p* < 0.05) (Table 4 and Fig. S1). Furthermore, the sensitivity increased by almost 10% (76.7% vs. 67.4%) (Table 4 and Fig. S1). Taken together, these findings demonstrate that compared to LSM, the combination of LSM, PLT, and Log_10_HBsAg can better predict the F ≥ 2 liver fibrosis stage, with a higher AUC and greater sensitivity.

**Table 4:** Improved performance of LPS for the identification of the F ≥ 2 fibrosis stage

Fibrosis stage	Cutoff	YI	Se (%, 95% CI)	Sp (%, 95% CI)	PLR (95% CI)	NLR (95% CI)	PPV (%, 95% CI)	NPV (%, 95% CI)	AUC (95% CI)
F ≥ 2	0.30	0.50	76.6 (67.6–84.1)	73.9 (58.9–85.7)	2.9 (1.8–4.8)	0.32 (0.20–0.50)	87.6 (81.2–92.1)	56.7 (47.3–65.6)	0.792 (0.720–0.852)

*LPS* (LSM, PLT and Log_10_ HBsAg) = 0.511 × LSM − 0.006 × PLT − 0.682 × Log_10_ HBsAg + 0.769, *Se* sensitivity, *YI* Youden index, *Sp* specificity, *PLR* positive likelihood ratio, *NLR* negative likelihood ratio, *PPV* positive predictive values, *NPV* negative predictive values, *AUC* area under receiver operating characteristics (ROC) curves, *CI* confidence interval

## Discussion

This study is the first to report that the LSM is a superior noninvasive index for predicting the HBV-related liver fibrosis stage in children aged 0–6 years, compared to APRI and FIB-4 scores. Furthermore, we found that the LSM was better able to distinguish the F0–F2 stage from the F3–F4 stage (AUC 0.941), compared to the F0–F1 and F2–F4 stages (AUC 0.732). This suggests that the LSM is particularly effective for the diagnosis of liver fibrosis in the F ≥ 3 stage.

While liver biopsies are currently the most commonly used test for the diagnosis of HBV-related fibrosis in children, its invasiveness limits its use in repeat assessments which are required for the dynamic monitoring of CHB development and the effects of antiviral treatment [8, 9]. Recently, a pediatric nonalcoholic steatohepatitis study reported TE AUCs of 0.992 and 1 for fibrosis stages F ≥ 2 and F ≥ 3, respectively; cut-off values for predicting the corresponding fibrosis stages were 7 kPa and 9 kPa, respectively [14]. Another study found that the 8.6 kPa cutoff point could be used to discriminate between stages F0–F2 and F3–F4 in children and young adults with multiple causes of liver disease [11]. In our study, we found that the AUCs were 0.732 and 0.941, and the cut-off values were 5.6 kPa and 6.9 kPa for fibrosis stages F ≥ 2 and F ≥ 3, respectively. The discrepancies between the findings of these studies may be due to differences in the age of the participants at the time of enrolment, as well as the causes of liver disease [19]. Consistent with our findings, the study by Anna et al. reported LSM of 5.4 (95% CI 4.0, 7.1) kPa for the F2 stage in children with CHC [20]. Moreover, a previous study demonstrated that the LSM was able to adequately predict the liver fibrosis stage in adult patients with CHB. The ROC curves were 0.81 for F0–F1 vs. F2–F4, and 0.93 for F0–F2 vs. F3–F4 [21], which are consistent with the results for children with CHB in our study. However, the cut-off values in adult patients with CHB were 7.2 kPa and 8.1 kPa for fibrosis stages F ≥ 2 and F ≥ 3, respectively [21]; this difference of the cut-off values between young children in our study and adult patients was also affected by subject age. Overall, the present study is the first to suggest that TE is a highly effective methodology for identifying advanced fibrosis (F ≥ 3) in children (aged 0–6 years) with CHB. Moreover, TE is vital for outpatient monitoring and clinical decision-making for children with CHB and advanced fibrosis, similarly to adults [22].

We additionally found that the APRI and FIB-4 did not provide additional advantages over the LSM for the discrimination of hepatic fibrosis stages F ≥ 2 and F ≥ 3. In agreement with some studies that have focused on adults with CHB [23, 24], we found that the APRI and FIB-4 were not suitable for predicting HBV-related fibrosis stages of F ≥ 2 and F ≥ 3 in CHB children. These results suggest that TE, APRI, and FIB-4 are suboptimal for the diagnosis of the F ≥ 2 stage. Previous studies have reported correlations between HBV/HCV-related fibrosis and the following factors: PLT count, log_10_HBsAg, alkaline phosphatase, ALT, AST, BMI, and inflammation [25–27]. Similarly, in our study, we found that LSM, PLT, and Log_10_HBsAg were independent factors associated with the F ≥ 2 fibrosis stage. This study is the first to combine these three independent factors to generate a “LPS” index and demonstrate an improvement in diagnostic accuracy for the F ≥ 2 fibrosis stage.

This study had several limitations. First, due to the low incidence of HBV-related advanced fibrosis in children, the number of patients with the F3–F4 fibrosis stage was small; this limited our ability to validate the cutoff points for identifying advanced fibrosis. Second, we only evaluated children aged 0–6 years, and our study was performed at a single center. Future studies should utilize larger sample sizes across multiple centers, to validate the cutoff points determined in this study. Furthermore, the performance of TE in the 7- to 18-year-old age group warrants consideration.

In conclusion, the LSM is a superior noninvasive index for the detection of LSM rather than APRI and FIB-4 offer excellent performance for children aged 0–6 years with HBV-related advanced fibrosis, compared to the APRI and FIB-4, in children aged 0–6 years in China. However, TE, APRI, and FIB-4 are suboptimal for the diagnosis of the F ≥ 2 fibrosis stage. The diagnosis of this stage can be significantly enhanced by the combination of LSM, PLT, and log_10_ HBsAg.

## Supplementary Information

Below is the link to the electronic supplementary material.

Supplementary file1 (DOC 43 KB)

Supplementary file2 (TIF 16671 KB)

## Abbreviations

AUC: Area under receiver operating characteristics (ROC) curves
ALT: Alanine aminotransferase
AST: Aspartate aminotransferase
ALP: Alkaline phosphatase
APRI: Aspartate aminotransferase (AST)-platelet ratio index (APRI)
HBeAg: Hepatitis B e-antigen
BMI: Body Mass Index
HBsAg: Hepatitis B surface antigen
CHB: Chronic hepatitis B
COI: Cut off index
CHC: Chronic hepatitis C
FIB-4: Fibrosis-4 index
IQR: Interquartile range
kPa: Kilopascal
LSM: Liver stiffness measurement
LPS index: (LSM, PLT and Log_10_HBsAg) = 0.511 × LSM − 0.006 × PLT − 0.682 × Log_10_HBsAg + 0.769
METAVIR: Meta-analysis of histological data in viral hepatitis
PLT: Platelet
PPV and NPV: Positive and negative predictive values
PLR and NLR: Positive and negative likelihood ratio
γ-GT: Gamma-glutamyl transpeptidase
TE: Transient elastography
WBC: White blood cell
