# Supplementary material for: Assessment of liver fibrosis by transient elastography in young children with chronic hepatitis B virus infection
Source: Hepatol Int. 2021 Jul 9;15(3):602–10. doi: 10.1007/s12072-021-10194-7 (PMC8286936; doi:10.1007/s12072-021-10194-7)
Supplement: Supplementary file 1 — Supplementary file1 (DOC 43 KB) [file 12072_2021_10194_MOESM1_ESM.doc]

**Table S1.** Demographic and clinical characteristics of children with CHB exhibiting fibrosis stages F < 2 and F ≥ 2

| **Variable** | **Fibrosis (n=157)** | | |
| --- | --- | --- | --- |
|  | **F** < **1 (n=111)** | **F ≥ 2 (n=46)** | ***P*** |
| Male gender, n (%) | 63 (56.8) | 29 (63.0) | 0.467 |
| Age (median, IQR, years) | 2.9 (1.9-4.3) | 3.1（2.2-3.1） | 0.512 |
| BMI (median, IQR, kg/m^2^) | 16.0 (15.0-17.5) | 16.0 (15.0-17.0) | 0.415 |
| ALT (median, IQR, IU/L) | 65 (39-126) | 108 (56-170) | 0.012 |
| AST (median, IQR, IU/L) | 69 (50-117) | 107 (68-154) | 0.001 |
| Total bilirubin (median, IQR, μmol/L) | 6.2 (5.0-7.9) | 6.2 (4.7-8.6) | 0.775 |
| ALP (median, IQR, IU/L) | 283 (235-332) | 277 (230-360) | 0.805 |
| γ-GT(median, IQR, IU/L) | 16 (12-26) | 25 (16-46) | 0.001 |
| Albumin (median, IQR, g/L) | 41 (39-43) | 41 (39-43) | 0.778 |
| Cholinesterase (median, IQR, IU/L) | 8165±1552 | 7374±1739 | 0.006 |
| WBC count (mean±SD, 10^9^/L) | 8.3±2.2 | 7.9±1.98 | 0.315 |
| PLT count (median, IQR, 10^9^/L) | 299 (251-349) | 247 (209-318) | 0.001 |
| HBeAg postitive, n (%) | 104 (93.7) | 39 (84.8) | 0.075 |
| HBeAg (median, IQR, COI) | 1541 (968-1879) | 1095 (59-1619) | 0.010 |
| HBsAg quantification (median, IQR, IU/mL) | 26744 (8033-52010) | 11255 (3002-22746) | <0.001 |
| Log_10_HBsAg (median, IQR, IU/mL) | 4.43 (3.90-4.72) | 4.05 (3.48-4.36) | <0.001 |
| Log_10_HBV DNA (median, IQR, IU/mL) | 7.92 (7.08-8.11) | 7.58 (6.84-8.00) | 0.020 |
| A≥2 | 57 (51.4%) | 41 (89.1%) | <0.001 |
| LSM (median, IQR, kPa) | 4.9 (4.3-5.6) | 6.2 (5.2-7.9) | <0.001 |

Notes: BMI, body mass index; ALT, alanine aminotransferase; AST, aspartate aminotransferase; ALP, alkaline phosphatase; γ-GT, gamma-glutamyl transpeptidase ; WBC, white blood cell; PLT, platelet; HBeAg, hepatitis B e-antigen; HBsAg, hepatitis B surface antigen; IQR, interquartile range; COI, cut off index; LSM, liver stiffness measurement.
