# Supplementary figures and images for: Assessment of liver fibrosis by transient elastography in young children with chronic hepatitis B virus infection
Source: Hepatol Int. 2021 Jul 9;15(3):602–10. doi: 10.1007/s12072-021-10194-7 (PMC8286936; doi:10.1007/s12072-021-10194-7)

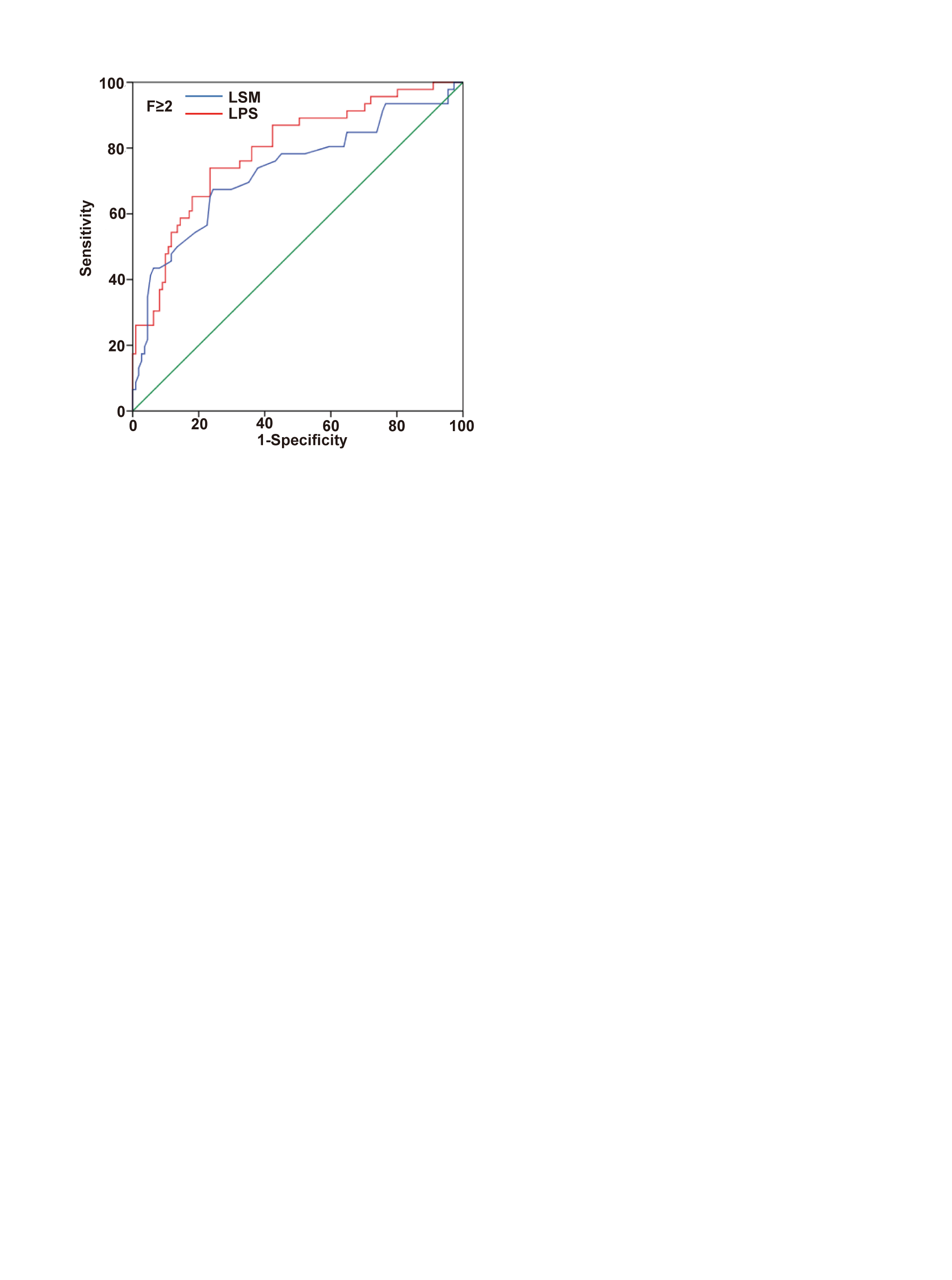

Supplement: Supplementary file 2 — Supplementary file2 (TIF 16671 KB) [file 12072_2021_10194_MOESM2_ESM.tif]
